# The impact of obesity and adiponectin signaling in patients with renal cell carcinoma: A potential mechanism for the “obesity paradox”

**DOI:** 10.1371/journal.pone.0171615

**Published:** 2017-02-08

**Authors:** Ryuichi Ito, Shintaro Narita, Mingguo Huang, Taketoshi Nara, Kazuyuki Numakura, Koichiro Takayama, Hiroshi Tsuruta, Atsushi Maeno, Mitsuru Saito, Takamitsu Inoue, Norihiko Tsuchiya, Shigeru Satoh, Tomonori Habuchi

**Affiliations:** 1 Department of Urology, Akita University Graduate School of Medicine, Akita, Japan; 2 AMED-CREST, Japan Agency for Medical Research and Development; 3 Department of Urology, Yamagata University Graduate School of Medicine, Yamagata, Japan; 4 Center for Kidney Disease and Transplantation, Akita University Hospital, Akita, Japan; Seoul National University College of Pharmacy, REPUBLIC OF KOREA

## Abstract

Although obesity increases the risk of renal cell carcinoma (RCC), obese patients with RCC experience longer survival than non-obese patients. However, the mechanism of this “obesity paradox” is unknown. We examined the impact of preoperative BMI, serum total adiponectin (sAd) level, total adiponectin secretion from perinephric adipose tissue, and intratumoral expression of adiponectin receptors on RCC aggressiveness and survival. We also investigated the mechanism underlying enhanced cancer aggressiveness in RCC cells stimulated with exogenous adiponectin. Overweight and obese patients had significantly lower grade cancers than normal patients in all patients and in those without metastasis (p = 0.003 and p = 0.027, respectively). Cancer-specific survival was significantly longer in overweight and obese patients than in normal patients in all patients (p = 0.035). There was a weak inverse correlation between sAd level and BMI in RCC patients (r = −0.344, p = 0.002). Tumor size was slightly correlated with sAd level, and high sAd was significantly associated with poor overall survival rates in patients with non-metastatic RCC (p = 0.035). Adiponectin levels in perinephric adipose tissue and intratumoral AdipoR1/R2 expression were not correlated with RCC aggressiveness or survival. Proliferation significantly increased in 786-O and Caki-2 cells exposed to exogenous adiponectin, whereas cell invasion and migration were unaffected. In addition, exogenous adiponectin significantly inhibited starvation- and metformin-induced apoptosis, and up-regulated p-AMPK and Bcl-xL levels. In summary, low BMI and high adiponectin levels are associated with aggressive cell behaviors and poor survival in surgically-treated RCC patients. The effects of adiponectin on proliferation and apoptosis might underlie the “obesity paradox” of RCC.

## Introduction

Obesity causes multiple health problems and is associated with an increased risk of death and an aggressive phenotype in several malignancies [1]. Previous studies have identified several mechanisms underlying obesity-related carcinogenesis, including the activation of growth factor signaling, the induction of specific lipids, and the secretion of various cytokines [2]. Although obesity is associated with an increased incidence of renal cell carcinoma (RCC) [3], obese patients with RCC experience longer survival than non-obese patients [4]. This phenomenon is known as the “obesity paradox”; however, the mechanisms underlying this phenomenon are poorly understood.

Adiponectin, a cytokine secreted from adipocytes, plays an important role in the progression of several cancers and obesity-related diseases [5]. Recent studies have demonstrated that low serum and plasma adiponectin levels in patients with RCC are associated with an aggressive phenotype and metastasis [6, 7]. Moreover, preclinical studies have demonstrated that exogenous adiponectin is capable of modulating cell proliferation and apoptosis in various biological processes, including cancer development [8, 9]. In addition, adiponectin receptor 1 (AdipoR1) and adiponectin receptor 2 (AdipoR2), the main regulators of adiponectin, have been implicated in carcinogenesis in several cancers, including RCC [10]. A growing body of evidence suggests that obesity and obesity-related cytokine signaling play important roles in RCC carcinogenesis.

In this study, we assessed the impact of obesity and adiponectin signaling on clinical outcomes in surgically-treated RCC patients using serum and tissue samples. Furthermore, we investigated the mechanism underlying the “obesity paradox” by examining the effect of exogenous adiponectin stimulation on tumorigenic activity in RCC cell lines.

## Materials and methods

### Patients

The Institutional Review Board of the Akita University School of Medicine, Akita, Japan approved all of the experiments, and human samples were collected after obtaining written informed consent. Our institutional Review Board approved the consent procedure, and consent was recorded in the patient’s medical record. A total 129 of patients with RCC who underwent kidney surgery between 2005 and 2011 at Akita University Hospital, Akita, Japan were included in the study. We excluded patients that did not provide written informed consent and/or for whom we did not obtain adequate samples. Patient characteristics are shown in Table 1. Serum total adiponectin levels in preoperative serum samples from 78 of the 129 patients were analyzed using a specific enzyme-linked immunosorbent assay (ELISA). Forty-one (31.8%) patients had undergone open kidney surgeries, and 88 (68.2%) patients had undergone endoscopic kidney surgeries. Radical nephrectomy and partial nephrectomy was conducted in 107 (82.9%) and 22 (17.1%) patients, respectively. Cytokine levels were measured in medium conditioned with perinephric adipose tissues harvested from patients with RCC who had undergone radical nephrectomy (n = 39) or from healthy controls that underwent living-donor nephrectomy between 2010 and 2014 (n = 40).

**Table 1:** Patient characteristics.

Variables		N = 129	%
Age (median, range)		68	22–93
Gender	Male	88	
	Female	41	
BMI (median, range)		23.1	16.0–40.9
BMI category	Normal	87	67.4
	Overweight	33	25.6
	Obese	8	6.2
	Unknown	1	0.8
Tumor location	Right	59	45.7
	Left	63	48.8
	Bilateral	7	5.4
Radiographic tumor size (cm, median, range)		4.5	0.3–15.0
Clinical T stage	1	84	65.1
	2	23	17.8
	3	19	14.7
	4	3	2.3
Clinical N stage	0	114	88.4
	1	15	11.6
Clinical M stage	0	111	86.0
	1	18	14.0
Surgical procedure	Open	41	31.8
	Endoscopic	88	68.2
Radicality	Radical	107	82.9
	Partial	22	17.1
Histology	Clear	114	88.4
	Non-clear	10	7.8
	With spindle	5	3.9
Fuhrman grade	1	5	3.9
	2	82	63.6
	3	37	28.7
	4	5	3.9
Pathologic T stage	1	87	67.4
	2	13	10.1
	3	27	20.9
	4	2	1.6
Pathologic N stage	0	87	67.4
	1–2	11	8.5
	x	31	24.0
Survival	Alive	114	88.4
	Dead	15	11.6

Body mass index (BMI) (kg/m^2^) was calculated as weight (kg) divided by height (m) squared. Patients were classified as normal weight (BMI < 25 kg/m^2^), overweight (BMI: 25–30 kg/m^2^), or obese (BMI ≥ 30 kg/m^2^) as previously described [1]. The tumor-node-metastasis (TNM) staging system was used to stratify patients by pathological stage [2], and nuclear grade was assigned according to Fuhrman's grading system [3].

### Serum and perinephric fat-secreted adiponectin levels

An ELISA kit specific for human total adiponectin (R&D Systems Inc., Minneapolis, MN) was used to measure adiponectin levels in serum and perinephric fat-conditioned medium according to the manufacturer’s instructions. Adipose tissue-conditioned medium was generated as previously described [4]. Briefly, adipose tissues were transferred to a Petri dish containing 20 ml of phosphate-buffered saline (PBS) and finely minced into 20- to 80-mg pieces using scissors. The tissue pieces were extensively washed with 200 ml of PBS using a filter with a pore size of 70 μm (BD Biosciences, San Jose, CA). Then, they were transferred to a 50 ml centrifuge tube containing 45 ml of 37°C PBS, and the tubes were gently shaken for 20 min. The tube contents were poured over a filter, and the tissue pieces were transferred to a tube containing 50 ml of PBS. Then, the tissue pieces were centrifuged for 1 min at 277 × g at room temperature to remove red blood cells and debris. Fat tissue was placed in a Petri dish with 10 ml of M199 culture medium (Invitrogen, Carlsbad, CA) supplemented with 50 μg/ml gentamicin. Medium collected 24 h later was separated into 1 ml aliquots and stored at −80°C.

### Quantitative reverse-transcription polymerase chain reaction (qRT-PCR)

mRNA levels of *AdipoR1* and *AdipoR2* in tumor tissues and surrounding normal renal tissue were measured using qRT- PCR. Total RNA was extracted from the tissues using TRIzol^™^ reagent (Invitrogen), and cDNA was synthesized using SuperScript^®^ II reagent (Invitrogen) according to the manufacturer’s instructions. The following primers were used: AdipoR1 forward 5′- AATTCCTGAGCGCTTCTTTCCT -3′ and reverse 5′- CATAGAAGTGGACAAAGGCTGC -3′, AdipoR2 forward 5′- TGCAGCCATTATAGTCTCCCAG -3′ and reverse 5′- GAATGATTCCACTCAGGCCTAG -3′, and β-actin forward 5′-ATCTGGCACCACACCTTCTA-3′ and reverse 5′-CGTCAT ACTCCTGCTTGCTGATCCACATCTGC-3′. The qRT-PCR conditions were as follows: 95°C for 30 s, 40 cycles of 95°C for 30 s and 60°C for 30 s, 95°C for 15 s, 60°C for 30 s, and 95°C for 15 s. Relative mRNA expression levels were determined using the 2-ΔΔCT method [5], and *β-actin* was used as an endogenous control. Each experiment was conducted in triplicate.

### Immunohistochemistry (IHC) analysis

Slides with tissue samples from 91 of 129 surgically-treated RCC patients were obtained from the Akita University Hospital. IHC staining was conducted as previously described [6]. Briefly, tissues were fixed in 20% formalin, embedded in paraffin, and sliced into 5-μm thick sections. The tissues were incubated with a rabbit polyclonal primary antibody against AdipoR1 (Abbiotec, San Diego, CA) diluted 1:200. The investigators that evaluated and scored the IHC assays (S.N. and M.H.) were blinded to the patient’s background and clinicopathological features. Membranous and/or cytoplasmic AdipoR1 staining intensity was classified as negative (0), weak (1), moderate (2), or strong (3). The percentage of positive cells of IHC staining was classified as 0% (0), <25% (1), 25%—<50% (2), 50%—<75% (3), 75%—100% (4). The immunoreactivity scores were determined by multiplying the intensity score by the percentage of positive cells.

### Cells and reagents

The human RCC cell lines (Caki-1, Caki-2, ACHN, and 786-O) and the murine RCC cell line RENCA were purchased from American Type Cell Culture Collection (Manassas, VA). The cells were cultured in RPMI 1640 (Invitrogen) supplemented with 10% fetal bovine serum (FBS) in a 5% CO_2_ humidified incubator at 37°C. Recombinant human Adiponectin/Acrp30 was purchased from R&D Systems (Minneapolis, MN). Metformin was purchased from Wako Pure Chemical Industries Ltd. (Osaka, Japan).

### Cell proliferation assay

786-O and Caki-2 cells were seeded into 96-well plates (3 × 10^3^ cells/well) in culture medium supplemented with 10% FBS. After a 24-h incubation in serum-free medium, the medium was replaced with medium supplemented with various doses of recombinant adiponectin for 24–48 h. Cell proliferation was analyzed using a non-radioactive 3-(4,5-dimethylthiazol-2-yl)-2,5-diphenyltetrazolium bromide (MTT)-based Cell Proliferation Kit (Roche Life Sciences, Branford, CT) according to the manufacturer’s instructions. Absorbance was measured using an ELISA reader (Bio-Rad Laboratories Inc., Hercules, CA). The proliferation assays were conducted in triplicate.

### Apoptosis analysis

Apoptosis was analyzed by staining the cells with propidium iodide (PI), either alone or in combination with Annexin V-fluorescein isothiocyanate (FITC), as previously described [7]. Briefly, 786-O and Caki-2 cells were serum-starved for 24 h. Then, the cells were cultured in the presence or absence of 250 ng/ml recombinant adiponectin and/or 20 mM metformin for 48 or 72 h. Based on the manufacturer’s instructions, PI staining and Annexin V/PI double staining assays were conducted using the CycleTEST PLUS DNA Reagent Kit (BD Biosciences) and the FITC Annexin V Apoptosis Detection Kit (BD Biosciences), respectively. The stained cells were analyzed using a FACSCalibur^™^ Cell Analyzer (BD Biosciences). Approximately 10000 cells per sample were examined. The data were analyzed using CellQuest^™^ software (BD Biosciences).

### Wound healing assay

The cells were seeded into a 35-mm dish (1 × 10^4^ cells/ml) with RPMI 1640 containing 5% FBS. A horizontal slit was made at the center of each well using a white tip. After 24 h, cell migration to the wound surface was analyzed under a light microscope. Each assay was conducted in triplicate.

### Matrigel^™^ invasion assay

The *in vitro* invasion assay was carried out in Growth Factor Reduced BD BioCoat^™^ Matrigel^™^ invasion chambers (BD Biosciences) according to the manufacturer’s instructions. Briefly, cells (1 × 10^4^) were seeded in serum-free medium in the upper compartment, and Dulbecco’s Modified Eagle’s Medium supplemented with 20% FBS was used a chemoattractant in the lower compartment of the chamber. After 22 h, non-invasive cells on the upper side of the chamber were removed, and the membranes were stained using a Diff-Quik Stain Kit (Sysmex Corp., Hyogo, Japan). Invasive capacity was quantitatively analyzed by measuring the number of invasive cells under a light microscope. Each assay was performed in triplicate.

### Western blot analysis

Total protein was isolated using complete Lysis-M buffer (Roche Diagnostics, Basel, Switzerland). Protein concentration was measured using a Nanodrop 1000 (Thermo Fisher Scientific, Waltham, MA). Equal amounts of protein lysates were separated using sodium dodecyl sulfate-polyacrylamide gel electrophoresis, and the separated proteins were transferred to a membrane using the iBlot^®^ Blotting System (Invitrogen). The membranes were blocked in Tris-buffered saline supplemented with 2% bovine serum albumin and 0.1% Tween-20. The membranes were incubated with diluted antibodies overnight and subsequently incubated with secondary IgG antibody for 1 h. The protein bands were detected using ECL Prime Western Blotting Detection Reagent (GE Healthcare, Buckinghamshire, UK). Anti-Bcl-2 and anti-Bcl-xL were purchased from Santa Cruz Biotechnology Inc. (Dallas, TX), and anti-β-actin, anti-AMPK, anti-p-AMPK, anti-AKT, anti-p-AKT, anti-MAPK, and anti-p-MAPK were purchased from Cell Signaling Technology Inc. (Danvers, MA).

### Statistical analysis

Statistical analyses were conducted using SPSS version 19 (IBM Corp., Armonk, NY). Differences in clinicopathological characteristics between each group were assessed using the Mann–Whitney U test, analysis of variance for continuous variables, and the χ^2^ test for categorical variables. Statistical differences among three groups were assessed using the Jonckheere–Terpstra test [8]. Cancer-specific survival (CSS) was defined as the time in months from the date of RCC diagnosis to RCC-related death. CSS and overall survival (OS) were determined using the Kaplan–Meier method and log-rank test. The multivariate analysis was conducted using a Cox proportional hazards regression model. Spearman tests were used to analyze correlations. The strength of the correlation was based on the correlation coefficient as follows: weak (0.1–0.3), moderate (0.3–0.5), or strong (>0.5). Differences with p-values <0.05 were considered statistically significant.

## Results

### Low BMI was associated with cancer aggressiveness and poor CSS in surgically-treated RCC patients

The median BMI of the 129 surgically-treated RCC patients was 23.1 kg/m^2^ (range: 16.0–40.9 kg/m^2^), with 25.6% (33/129) of patients classified as overweight and 6.2% (8/129) classified as obese. First, we investigated the relationship between BMI and clinicopathological outcomes (Table 2, S1 Table). There was no significant difference in the proportion of patients with non-clear cell RCC between the normal weight and overweight/obese group (Table 2, S1 Table; p = 0.362). Regarding the relationship between RCC aggressiveness and BMI, overweight/obese patients had a lower incidence of large tumor size (based on radiographic assessment) and a lower pathologic stage normal weight patients; however, these differences were not statistically significant (Table 2, S1 Table). Conversely, overweight/obese patients had significantly lower grade cancer than normal weight patients (Table 2, S1 Table; p < 0.001).

**Table 2:** The relationship between BMI and clinicopathologic outcomes in patients with RCC treated with renal surgery.

		BMI category (%)		p value
		Normal	Overweight and obese	
Histology	Clear	81(91.0)	37 (94.9)	0.362
	Non-clear	8 (9.0)	2 (5.1)	
Radiographic tumor size	< 7cm	63 (70.8)	29 (74.4)	0.425
	≥ 7cm	26 (29.2)	10 (25.6)	
Pathologic T stage	1–2	67 (75.3)	33 (84.6)	0.173
	3–4	22 (24.7)	6 (15.4)	
Fuhrman grade	1–2	53 (59.6)	34 (87.2)	< 0.001
	3–4	36 (40.4)	5 (12.8)	

**Fig 1:**
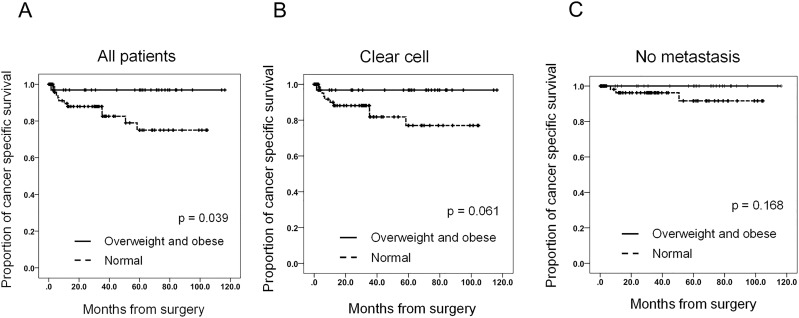
BMI affects CSS in patients with surgically-treated RCC. A–C. BMI is associated with CSS in patients with surgically-treated RCC. CSS in normal and overweight/obese patients (A), patients with clear cell RCC (B) and patients with non-metastatic RCC (C).

Fifteen (11.6%) patients died during the median follow-up period of 30.2 months, and 13 (10.1%) of these deaths were due to RCC progression. In the overall patient population, CSS was significantly longer in overweight/obese patients compared with normal weight patients (Fig 1A, S1 Table; p = 0.039). The 5-year CSS in normal and overweight/obese patients was 75% and 96.9%, respectively. Among patients with clear cell RCC, CSS tended to be longer in overweight/obese patients compared with normal weight patients (Fig 1B, S1 Table; p = 0.061). However, BMI was not significantly associated with CSS in the group of patients with non-metastatic RCC (Fig 1C, S1 Table; p = 0.168) or in the low grade tumor (p = 0.331) or high grade tumor groups (p = 0.368) (S1 Fig, S1 Table). In a univariate analysis, low BMI (<23.1), larger tumor size, high clinical T stage, high clinical M stage, high pathologic T stage, high pathologic N stage, and high Furman grade were significantly associated with poor CSS (Table 3, S1 Table). A multivariate analysis using a Cox proportional hazard regression model revealed that low BMI, clinical M stage, and pathologic N stage were independent prognostic factors for poor CSS in the overall population of patients with surgically-treated RCC (Table 3, S1 Table). We also conducted multivariate analyses after adjusting each confounder which was significantly associated with CSS in the univariate analysis. After adjusting the analysis for tumor size, clinical T stage, pathologic T stage, and pathologic N stage, low BMI was an independent prognostic factor of CSS; however, the statistical significance of the association diminished after adjusting for clinical M stage and Furman grade (S2 and S1 Tables). These results suggested that low BMI is associated with an aggressive RCC phenotype and poor outcomes in patients with surgically-treated RCC.

**Table 3:** Cox proportional hazard for CSS in patients with RCC who underwent renal surgery.

	Univariate			Multivariate (stepwise)		
	HR	95% CI	p value	HR	95% CI	p value
Sex (male vs. female)	0.548	0.184–1.634	0.281			
Age (continuous)	1.005	0.960–1.053	0.822			
BMI (overweight/obese vs normal)	0.153	0.020–1.184	0.072			
BMI (< median = 23.1 vs ≥ median)	7.058	1.557–32.002	0.011	35.064	3.422–359.303	0.003
Radiographic tumor size (≥7cm vs <7cm)	4.431	1.445–13.592	0.009			
Clinical T stage (≥ 3 vs ≤ 2)	8.120	2.649–24.891	<0.001			
Clinical M stage (1 vs 0)	27.388	7.425–101.028	<0.001	10.141	2.338–43.978	0.002
Pathologic T stage (≥ 3 vs ≤ 2)	8.858	2.719–28.857	<0.001			
Pathologic N stage (1–2 vs 0 and x)	33.372	10.570–105.362	<0.001	46.117	6.694-317-732	<0.001
Fuhrman grade (≥ 3 vs ≤ 2)	35.474	4.583–274.590	0.001			
Histology (clear vs non-clear)	1.847	0.408–8.356	0.426			

### Serum total adiponectin levels are inversely correlated with BMI and directly correlated with aggressive RCC behaviors and poor overall survival in patients with RCC

**Fig 2:**
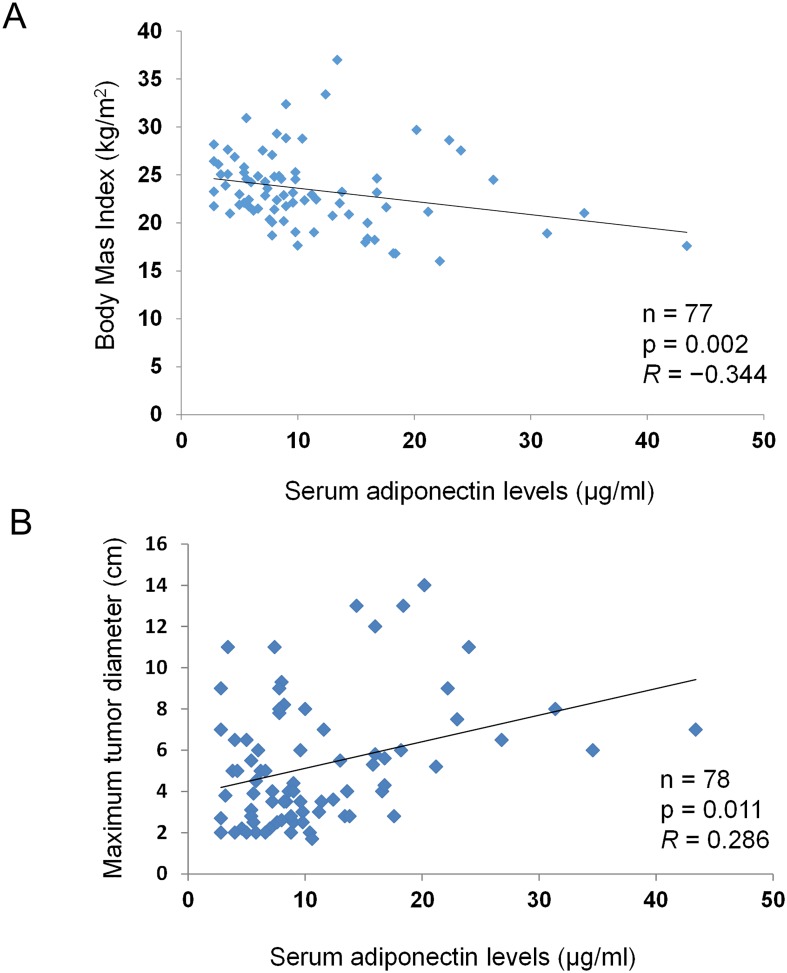
The relationship between serum total adiponectin, BMI, and RCC aggressiveness. The correlation between serum total adiponectin and clinical variables were analyzed using Spearman tests. Adiponectin level was inversely correlated with BMI (A) and directly correlated with radiographically-assessed tumor size (B). A correlation coefficient of 0.1–0.3 was considered weak, 0.3–0.5 was considered moderate, and >0.5 was considered strong.

Next, we assessed the association of presurgical serum total adiponectin level with BMI and clinicopathological features of RCC patients. Mean serum total adiponectin level in the overall patient population was 8.8 μg/ml (range: 2.8–43.4 μg/ml). Serum total adiponectin level was inversely correlated with BMI (Fig 2A, S1 Table; r = − 0.344, p = 0.002) and the tumor diameter on radiographic assessment was slightly correlated with the serum total adiponectin level (Fig 2B, S1 Table; r = 0.286, p = 0.011). Regarding the relationship between presurgical serum total adiponectin level and clinical variables, mean serum total adiponectin level was significantly greater in patients with tumors ≥ 4 cm (13.12 μg/ml) than patients with tumors < 4 cm (8.17 μg/ml) (Table 4, S1 Table; p = 0.001). and patients with a tumor size had a tendency toward a higher Mean serum total adiponectin level was also higher in patients with tumors ≥ 7 cm (14.53 μg/ml) compared with patients with tumors < 7 cm (9.82 μg/ml); however, the difference was not statistically significant (Table 4, S1 Table; p = 0.069). There was no significant association between mean serum total adiponectin level and other clinicopathological features, including histologic subtype, Fuhrman grade, pathologic T stage, clinical N, clinical M (Table 4, S1 Table).

**Table 4:** The relationship betweem serum total adiponectin levels and clinicopathological outcome in patients with RCC who underwent renal surgery.

Variables	No. of the patient	Serum total adiponectin level (ug/ml)		p value
		Mean	SD	
Histology				
clear	74	11.02	7.73	0.623
non-clear	1	17.60		
spindle	3	9.00	2.08	
Radiographic tumor size				
<4cm	33	8.17	3.4	0.001
≥4cm	45	13.12	9.03	
<7cm	58	9.82	5.94	0.069
≥7cm	20	14.53	10.48	
Fuhrman grade				
1+2	55	10.31	7.91	0.197
3+4	23	12.75	6.58	
Pathologic T stage				
1+2	62	10.69	8.04	0.441
3+4	16	12.34	5.47	
Clinical N stage				
0	71	11.10	7.86	0.789
1+2	7	10.29	4.13	
Clinical M stage				
0	69	11.10	7.91	0.816
1	9	10.48	4.56	

**Fig 3:**
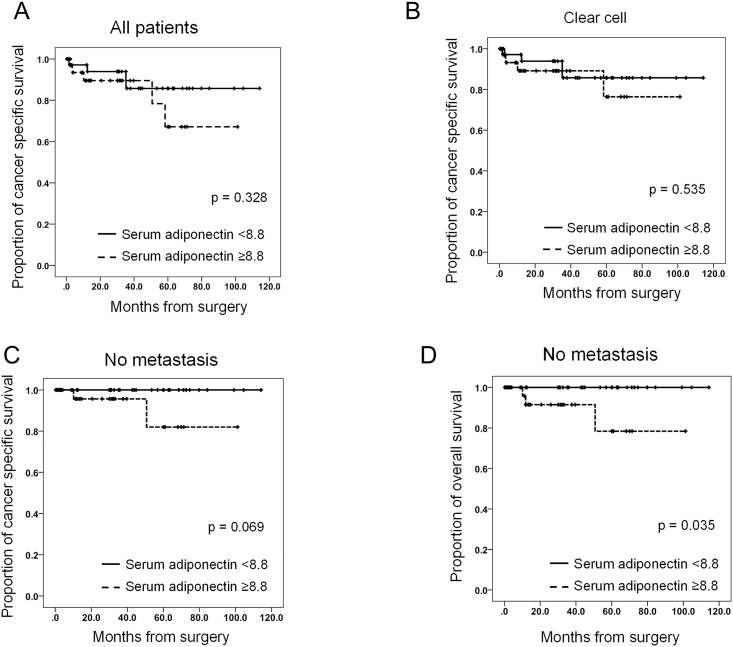
The relationship between serum total adiponectin level and survivals in patients with surgically-treated RCC. A–D. Serum total adiponectin level is associated with CSS and OS in patients with surgically-treated RCC. Subgroup analysis of CSS in patients stratified by serum total adiponectin level (A), patients with clear cell RCC (B), and patients with non-metastatic RCC (C). Analysis of OS in patients with non-metastatic RCC stratified by serum total adiponectin level (D)

We also conducted a subgroup analysis of CSS in surgically-treated RCC patients stratified by serum total adiponectin levels. The patients were divided into two groups based on the median serum total adiponectin level (8.8 μg/ml). In the group of patients with clear cell carcinoma, there was no significant difference in CSS in patients with serum adiponectin ≥ 8.8 μg/ml and patients with serum adiponectin < 8.8 μg/ml (Fig 3A and 3B, S1 Table). However, when the analysis focused on patients without metastasis, the CSS tended to be lower in patients with serum adiponectin ≥ 8.8 compared with patients with serum adiponectin < 8.8 (Fig 3C, S1 Table; p = 0.069). In addition, OS significantly decreased in patients with serum adiponectin ≥ 8.8 μg/ml compared with those with serum adiponectin < 8.8 μg/ml (Fig 3D, S1 Table; p = 0.035). Collectively, these results indicate that lower BMI was associated with higher serum total adiponectin, and a higher serum total adiponectin was potentially associated with cancer progression. In addition, after adjusting the continuous serum total adiponectin levels, low BMI was significantly associated with poor CSS (S1 Table; hazard ratio [HR]: 5.201, 95% confidence interval [CI]: 1.039–26.043, p = 0.045).

Next, we analyzed the relationship between mean total adiponectin level in perinephric adipose tissue-conditioned medium and the clinicopathological features of surgically-treated RCC patients (Table 5, S3 Table), as adipocytes are the primary source of plasma adiponectin in humans [9]. There were no significant differences in the mean adiponectin levels in perinephric adipose tissue-conditioned medium from control individuals (living healthy donors) and patients with RCC (Table 5, S3 Table; p = 0.115), whereas mean total adiponectin in perinephric adipose tissue-conditioned medium tended to be lower in surgically-treated RCC patients with a high BMI (Table 5, S3 Table; p = 0.088, Jonckheere–Terpstra test). Regarding the association between total adiponectin levels in perinephric adipose tissue and the clinicopathological features of RCC patients, mean total adiponectin in perinephric adipose tissue-conditioned medium was not associated with pathologic T stage or Fuhrman grade (Table 5, S3 Table). These results indicated that local adiponectin secretion from perinephric adipose tissue might be associated with BMI in patients with RCC; however, it does not appear to affect RCC aggressiveness and survival.

**Table 5:** Total adiponectin levels in perinephric fat-conditioned medium in patients with RCC and healthy controls who underwent renal surgery.

Variables	No. of the patient	Mean adiponectin level (ug/ml)	SD	p value
BMI category				
Normal	44	203.34	89.8	0.088*
Overweight	29	177.55	83.39	
Obese	5	149.22	70.81	
Patient				
Control	40	204.11	75.77	0.115
RCC	38	175.72	96.23	
Pathologic T stage				
1+2	31	181.60	93.05	0.436
3+4	7	149.72	113.33	
Fuhrman grade				
1+2	27	182.32	92.79	0.516
3+4	11	159.56	107.08	

*p value for trend; Jonckheere–Terpstra test

### *AdipoR1* and *AdipoR2* expression in human RCC tissues

**Fig 4:**
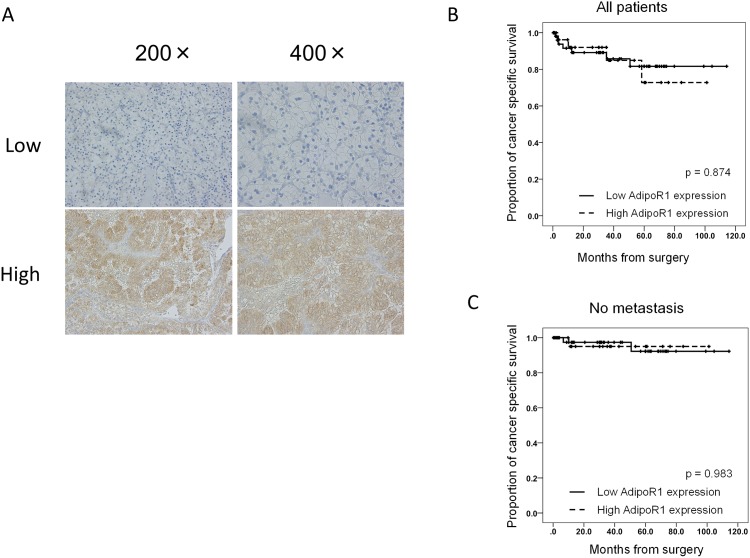
AdipoR1 protein levels in human RCC tissues. An immunoreactivity score > 4 was defined as high AdipoR1 levels, and an immunoreactivity score ≤4 was defined as low AdipoR1 levels. A Representative images of low and high AdipoR1 levels in human RCC epithelial tissue. AdipoR1 immunoreactivity score was not significantly associated with CSS in the overall RCC patient population (B) and in patients with non-metastatic RCC (C).

Two adiponectin receptors, AdipoR1 and AdipoR2, have been cloned [10]. We investigated the association between *AdipoR1* and *AdipoR2* expression and RCC aggressiveness and survival. Consistent with a previous study [11], the *AdipoR1* and *AdipoR2* expression levels significantly decreased in tumor tissues compared with surrounding normal parenchyma tissue (Table 6, S1 Table). In the subgroup analyses, pathological features of RCC were not associated with *AdipoR1* and *AdipoR2* expression levels (Table 6, S1 Table). AdipoR1 is ubiquitously expressed in obesity-related cancers [12]. Therefore, we assessed AdipoR1 protein levels in specimens obtained from surgically-treated RCC patients using immunohistochemistry. AdipoR1 predominantly localized to the membrane and cytoplasm of cancer cells, and representative images of low and high expression of adipoR1 are shown in Fig 4A. As shown in Table 7 and Fig 4B and 4C, AdipoR1 expression was not associated with cancer aggressiveness, clinicopathological features, or survival in patients with surgically-treated RCC. These results suggest that the impact of obesity-associated adiponectin signaling on RCC aggressiveness is not associated with the expression of adiponectin receptors.

**Table 6:** The mRNA levels of adiponectin receptors in tissues extracted from patients with RCC who underwent renal surgery.

		n	mRNA expression of *adipoR1* (relative to *β-actin*)			mRNA expression of *adipoR2* (relative to *β-actin*)		
			Mean	SD	p value	Mean	SD	p value
Normal parenchyma		110	0.077	0.031	<0.001	0.014	0.001	<0.001
Cancerous tissue		111	0.044	0.022		0.006	0.001	
Histological type	Clear	99	0.043	0.021	0.401	0.009	0.003	0.464
	Non-clear	12	0.051	0.032		0.008	0.006	
Pathologic T stage	1+2	83	0.044	0.022	0.421	0.010	0.007	0.322
	3+4	27	0.040	0.018		0.008	0.005	
Fuhrman grade	1+2	73	0.045	0.022	0.082	0.010	0.007	0.276
	3+4	37	0.078	0.018		0.008	0.006	

**Table 7:** The relationship between AdipoR1 expression and clinicopathological outcomes by immunohistochemistry.

Variables	AdipoR1 expression		p value
	low (0–4)	high (6–12)	
Sex			
Male	38	25	0.236
Female	21	7	
Age			
≤median	31	19	0.660
>median	28	13	
BMI			
Normal	43	25	0.388
Overweght+obesity	16	7	
Histology			
Clear	52	25	0.379
Non-clear	4	5	
Spindle	3	2	
Radiographic tumor size			
<4cm	25	13	1.000
≥4cm	34	19	
<7cm	45	23	0.801
≥7cm	14	9	
Fuhrman grade			
1+2	42	19	0.350
3+4	17	13	
Pathological T stage			
1+2	45	25	1.000
3+4	14	7	
Clinical N stage			
0	53	28	0.715
1+2	6	4	
Clinical M stage			
0	52	27	0.747
1	7	5	

### Exogenous adiponectin enhanced RCC cell proliferation

**Fig 5:**
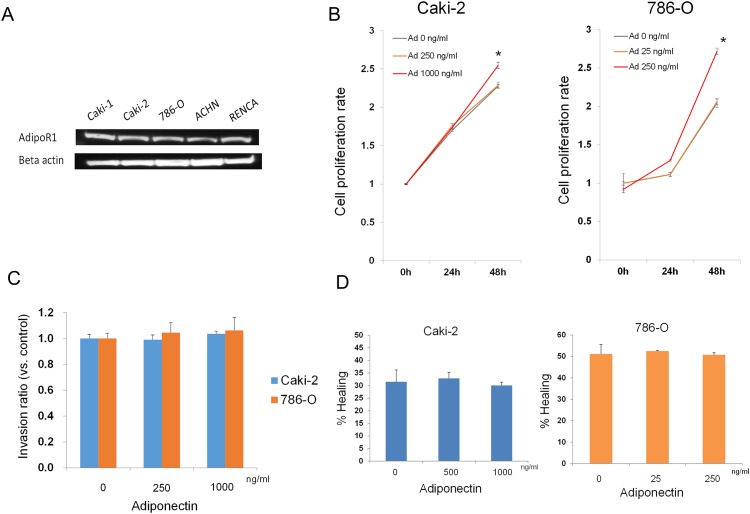
The effect of exogenous adiponectin on proliferation, invasion, and migration in 786-O and Caki-2 RCC cells. A. Adiponectin receptor 1 (AdipoR1) (upper) and β-actin (lower) protein levels in Caki-1, Caki-2, 786-O, ACHN, and RENCA cells were determined using western blot. AdipoR1 was detected in all cell lines. B. Caki-2 and 786-O cells were treated with various concentrations of recombinant adiponectin (Ad) (25–1000 ng/mL) and analyzed using an MTT assay 0, 24, and 48 h after adiponectin stimulation. *p < 0.05 compared with cell viability at 0 h. C. The histogram presents the invasive capacity of Caki-2 and 786-O cells treated with 250 or 1000 ng/ml recombinant adiponectin. The number of invasive cells in adiponectin-treated cells was compared with the number of invasive cells at 0 h (untreated cells). D. Caki-2 and 786-O cells were stimulated with 250–1000 ng/ml recombinant adiponectin. Cell migration to the wound surface was analyzed under a light microscope. The diameter of the wound in Caki-2 and 786-O cells treated with various concentration of recombinant adiponectin for 24 h was compared with the diameter of the wound in untreated cells.

To identify the mechanism underlying adiponectin-enhanced cancer aggressiveness, we conducted *in vitro* functional analyses using RCC cell lines. All of the RCC cell lines used in this study expressed AdipoR1 (Fig 5A, S2 Fig). We evaluated the effect of adiponectin stimulation on proliferation, invasion, and migration in 786-O and Caki-2 human RCC cells. In an MTT assay, the rate of proliferation significantly increased in Caki-2 and 786-O cells treated with recombinant adiponectin for 48 h (Fig 5B, S4 Table; p = 0.021 and p = 0.003, respectively). In contrast, there were no significant changes in invasion and migration in Caki-2 and 786-O cells treated with various concentrations of recombinant adiponectin (Fig 5C and 5D, S5 and S6 Tables). Collectively, these results indicate that exogenous adiponectin enhances RCC cell proliferation but does not affect invasion or migration *in vitro*.

### Adiponectin inhibited starvation- and metformin-induced apoptosis, and upregulated proteins related to cell growth and apoptosis *in vitro*

**Fig 6:**
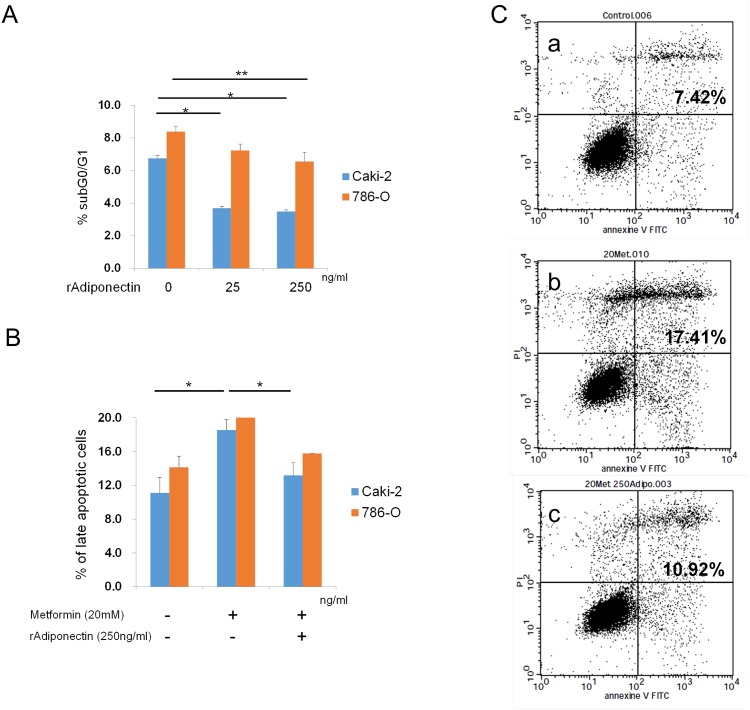
The effect of adiponectin on apoptosis in Caki-2 and 786-O cells. A. Flow cytometry analysis of PI-stained cells was used to evaluate the effect of recombinant adiponectin stimulation on serum starvation-induced apoptosis in Caki-2 and 786-O cells. The histogram shows the mean sub-G0/G1 fractions from two separate samples of Caki-2 and 786-O cells stimulated with adiponectin (0–250 ng/ml). Each bar represents the mean ± standard error. *p < 0.05. **p < 0.01. B and C. Flow cytometry analysis of Annexin V/PI-stained cells was used to analyze the effect of adiponectin stimulation on metformin-induced apoptosis in 786-O and Caki-2 cells. 786-O and Caki-2 cells were treated with 250 ng/ml recombinant adiponectin and 20 mM metformin, and subsequently harvested for flow cytometry. The histogram presents the mean percentage of cells in late apoptosis (B). *p < 0.05. C. Representative images of Annexin V/PI-stained 786-O cells incubated in the presence or absence of adiponectin and metformin (a. untreated cells b. cells treated with metformin alone, c. cells treated with metformin and adiponectin).

To identify the potential mechanism by which recombinant adiponectin enhances RCC cell proliferation, we evaluated apoptosis and the modulation of proteins associated with apoptosis and proliferation in 786-O and Caki-2 cells treated with recombinant adiponectin. Exogenous adiponectin significantly reduced the percentage of serum-starved cells in the sub-G0/G1 fraction in a dose-dependent manner (Fig 6A, S7 Table). Metformin, a commonly-used antidiabetic drug that modulates glucose and fatty acid metabolism, is known to induce cell cycle arrest and apoptosis in 786-O and Caki-2 cells [13]. Therefore, we investigated the impact of adiponectin stimulation on metformin-induced apoptosis in 786-O and Caki-2 cells (Fig 6B and 6C, S8 Table). As expected, the percentage of cells in late apoptosis significantly increased in 786-O and Caki-2 cells treated with 20 mM metformin. Interestingly, recombinant adiponectin significantly abrogated metformin-induced apoptosis in 786-O and Caki-2 cells (Fig 6B and 6C, S8 Table). These results suggest that exogenous adiponectin inhibits starvation- and metformin-induced apoptosis in RCC cells.

**Fig 7:**
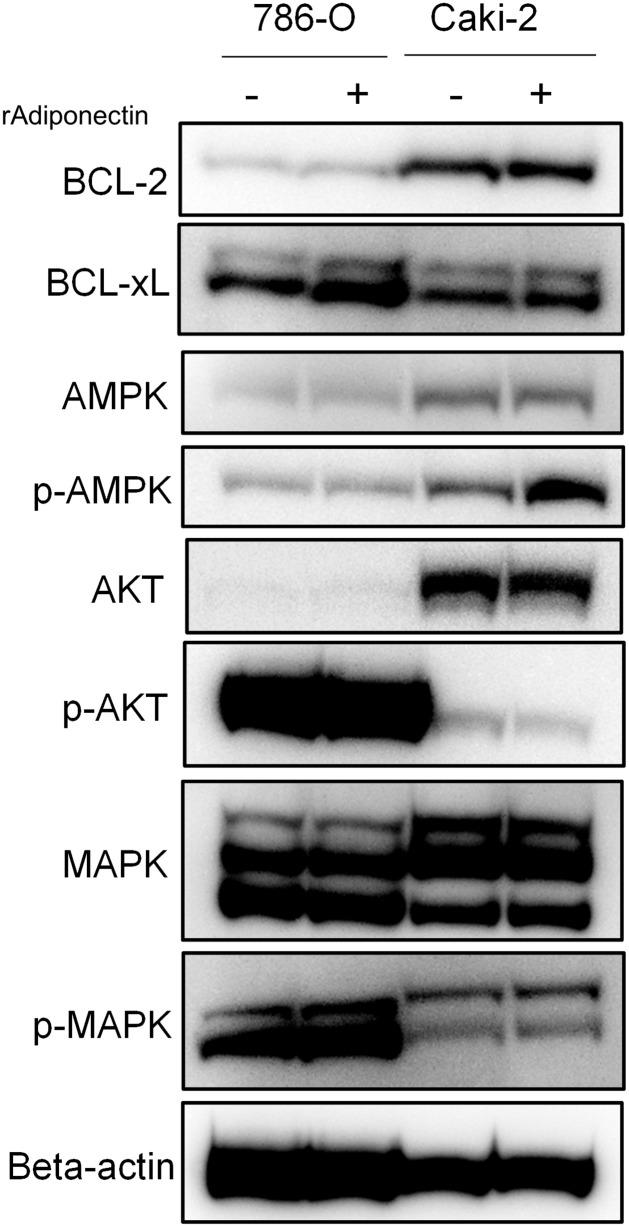
The effect of adiponectin on the expression of apoptosis- and proliferation-related proteins in Caki-2 and 786-O cells. Candidate downstream targets of exogenous adiponectin stimulation (250 ng/ml in 786-O cells and 1000 ng/ml in Caki-2 cells). After 72 h of adiponectin stimulation, Bcl-2, Bcl-xL, AMPK, p-AMPK, AKT, p-AKT, MAPK, p-MAPK, and β-actin levels were evaluated using western blot.

To identify the effectors of adiponectin-induced apoptosis in 786-O and Caki-2 cells, we analyzed levels of apoptosis- and proliferation-related proteins in 786-O and Caki-2 cells treated with and without recombinant adiponectin (Fig 7, S3 Fig). We found that p-AMPK was overexpressed in Caki-2 cells and Bcl-xL was overexpressed in 786-O cells stimulated with 250 nM recombinant adiponectin compared with untreated cells (Fig 7). Therefore, we speculate that adiponectin enhances proliferation by inhibiting apoptosis, and that the effect of adiponectin on proliferation and apoptosis in RCC cells is mediated by cell-specific targets.

## Discussion

In this study, we demonstrated that serum total adiponectin level is inversely correlated with BMI, low BMI and high serum total adiponectin level are associated with RCC aggressiveness and poor survival, and adiponectin secretion from perinephric fat tissue and intratumoral *AdipoR1*/*R2* expression are not associated with RCC outcomes. Furthermore, we demonstrated that exogenous adiponectin enhanced cancer cell proliferation *in vitro* by inhibiting apoptosis with modulating protein kinase and apoptosis-related protein activity. These observations might underlie the phenomenon known as the “obesity paradox” in RCC.

In a large, single-institution clinical study of 2119 US patients with surgically-treated clear cell RCC, obese and overweight patients were less likely to present with advanced stage and advanced grade cancers compared with normal weight patients (odds ratios of 0.61 and 0.73, respectively) [1]. In this study, we demonstrated that overweight and obese Japanese patients had significantly lower-grade cancers than normal weight patients. In addition, Choi et al. identified a statistically significant inverse correlation between BMI and CSS (HR = 0.47; 95% CI = 0.29–0.77) that persisted in a multivariate analysis adjusted for tumor stage, tumor size, tumor grade, symptoms, and baseline weight loss [14]. Consistent with these results, we found that CSS was significantly longer in overweight and obese patients compared with normal weight patients in a cohort of Japanese patients with RCC. Choi et al. also conducted a meta-analysis of 14 studies and demonstrated that high BMI was associated with significantly longer CSS (pooled HR = 0.59, 95% CI = 0.48–0.74) [14]. Furthermore, in a large, prospective, international study of patients with high-risk clear cell RCC by Donin et al., a multivariate analysis demonstrated that obese (BMI: ≥ 30) or morbidly obese (BMI: ≥ 35) patients had a statistically significant OS advantage [15]. Our findings confirm those of previous studies on the “obesity paradox” in patients with RCC, especially in Asian patients.

Several studies regarding the relationship between serum adiponectin levels and RCC aggressiveness, including a Japanese series of studies, have been reported. Horiguchi et al. demonstrated that serum levels of total and high molecular weight (HMW) adiponectin in RCC patients with metastasis were significantly lower compared with patients with non-metastatic RCC [16]. Martino et al. screened 301 consecutive patients with RCC to evaluate biomarkers associated with lipid metabolism, and RCC pathology and survival [17]. In contrast with our results, they found that patients with lower adiponectin levels had more aggressive tumors and lower survival rates. Although the precise explanation for these discrepancies remains unclear, circulating adiponectin level are known to be affected by multiple factors, including diabetes, racial background, cardiovascular disease, hypertension, and circadian rhythmicity [18] [19]. Although both the clinical and *in vitro* results of the present study demonstrated that adiponectin enhances cancer aggressiveness, a well-organized prospective study is needed to validate our results.

As recent studies have shown that cytokine stimulation from periprostatic adipose tissues enhances cancer aggressiveness in patients with prostate cancer [4] [20], we hypothesized that adiponectin secretion from perinephric adipose tissue modulates RCC aggressiveness by modulating the tumor microenvironment. However, adiponectin levels in perinephric fat-conditioned medium were not significantly associated with RCC aggressiveness, whereas adiponectin levels in clinical samples tended to be associated with BMI. Our results suggest that there might be a stronger association between RCC aggressiveness and systematic adiponectin levels compared with local adiponectin levels in adipose tissue surrounding the kidney, whereas local adiponectin levels had the potential to reflect systemic obesity.

AdipoR1 and AdipoR2, the main receptors for the adiponectin ligand [10], have been cloned. AdipoR1 and adipoR2 expression was not associated with RCC aggressiveness and survival in this study. A recent study demonstrated that adiponectin signaling was partly enhanced by an another ligand, T-cadherin [21]. T-cadherin also strongly binds HMW adiponectin and its localization pattern is similar to that of adiponectin receptors [21]. Indeed, T-cadherin has been shown to regulate the progression of several types of cancers, including breast, hepatic, and skin cancer, by modulating tumor cell proliferation and migration, and influencing intratumoral angiogenesis [22]. It will be intriguing to elucidate the role of T-cadherin on the interrelationship between obesity, adiponectin, and RCC; however, the role of T-cadherin in RCC remains unknown.

Previous studies on the impact of adiponectin stimulation on the proliferation of various types of cancer cells have generated conflicting results [23]. Evidence suggests that exogenous adiponectin inhibits cancer cell proliferation [23]. In contrast, Ogunwobi et al. demonstrated that full-length and globular adiponectin induces HT-29 colon epithelial cell proliferation and the secretion of several pro-proliferative and pro-inflammatory cytokines [24]. Furthermore, Lee et al. demonstrated that the upregulation of adiponectin and AdipoR1 enhances HEK293 cell growth *in vitro* by modulating the Ras-ERK1/2 pathway [25]. Adiponectin is known to play both pro- and anti-apoptotic roles in many fundamental processes and diseases [26]. In this study, adiponectin promoted cancer aggressiveness by enhancing proliferation and inhibiting apoptosis *in vitro*. However, the dose- and cell-dependent mechanisms mediating the effects of exogenous adiponectin stimulation in preclinical models of RCC merit further investigation.

We found that AMPK, an established regulator of adiponectin-mediated apoptosis inhibition [27], is activated by adiponectin stimulation in Caki-2 cells. Shibata et al. demonstrated that adiponectin inhibits apoptosis in cultured cardiac cells and that this effect was antagonized by dominant negative AMPK [27]. However, the precise role of AMPK in cancer is controversial, as AMPK is known to be function as both a tumor suppressor and an oncogene [28]. In addition, the Bcl-2-related gene *Bcl-xL* is upregulated in adiponectin-stimulated 786-O cells. Gobe et al. investigated the interrelationship between the expression of anti-apoptotic proteins such as Bcl-xL, the incidence of apoptosis, and mitosis in RCC biopsy specimens [29]. They found that *Bcl-xL* was overexpressed and apoptosis activity was minimal in specimens from patients with progressive, treatment-resistant RCC. Regarding other downstream effectors of adiponectin in RCC cells, Chou et al. revealed that adiponectin activates several signaling pathways, including the STAT3, S6, ERK1/2, and AKT pathways, in a dose-dependent manner in 786-O cells [12]. We were unable to identify a common downstream target of adiponectin stimulation *in vitro*. However, this might have been partly due to the distinct characteristics, such as von Hippel–Lindau gene status, in the two RCC cell lines used in this study [30].

This study has several limitations. First, we did not assess the distinct impact of different adiponectin isoforms. Second, the interaction between adiponectin and insulin signaling was not considered. This interaction might be associated with RCC aggressiveness, as adiponectin knockout mice develop insulin resistance and accumulating evidence in humans indicates that adiponectin plays an important role in insulin dynamics. Third, due to the retrospective nature of this study, we were unable to obtain a complete set of samples from the participants. In addition, the duration of the follow-up period was relatively short. Therefore, a study with a longer follow-up period and additional subgroup analyses are required to verify our findings. Furthermore, we should utilize additional approaches to investigate the complex relationship between obesity, adiponectin, and RCC incidence/severity.

In conclusion, BMI was inversely correlated with serum adiponectin level in RCC, and low BMI and high adiponectin might be associated with aggressive clinicopathological features and poor survival in surgically-treated RCC patients. Adiponectin stimulation promoted proliferation, inhibited apoptosis, and modulated levels of several target proteins. Although the interpretation of the results was complicated and we cannot extrapolate these results to clinical practice, insight into the “obesity paradox” can help facilitate the development of precision medicine and novel therapeutics for RCC.

## Supporting information

S1 Fig The impact of BMI on CSS in patients with low or high grade tumors. (TIF)

S2 Fig Gel images of the western blot analysis shown in Fig 5. (TIF)

S3 Fig Gel images of the western blot analysis shown in Fig 7. (TIF)

S1 Table The impact of BMI, serum total adiponectin levels, *AdipoR1/R2* expression, and AdipoR1 protein levels on RCC aggressiveness and patient survival. (XLS)

S2 Table Adjusted multivariate analyses of CSS in patients with surgically-treated RCC. (XLSX)

S3 Table Adiponectin levels in medium conditioned with perinephric adipose tissue obtained from surgically-treated RCC patients. (XLS)

S4 Table Analysis of proliferation in RCC cell lines treated with exogenous adiponectin. (XLSX)

S5 Table Analysis of invasion in RCC cell lines treated with exogenous adiponectin. (XLSX)

S6 Table Analysis of migration in RCC cell lines treated with exogenous adiponectin. (XLSX)

S7 Table The relationship between starvation-induced apoptosis and exogenous adiponectin stimulation in RCC cells. (XLS)

S8 Table The relationship between metformin-induced apoptosis and exogenous adiponectin stimulation in RCC cell lines. (XLS)
