# Supplementary figures and images for: The impact of obesity and adiponectin signaling in patients with renal cell carcinoma: A potential mechanism for the “obesity paradox”
Source: PLoS One. 2017 Feb 8;12(2):e0171615. doi: 10.1371/journal.pone.0171615 (PMC5298294; doi:10.1371/journal.pone.0171615)

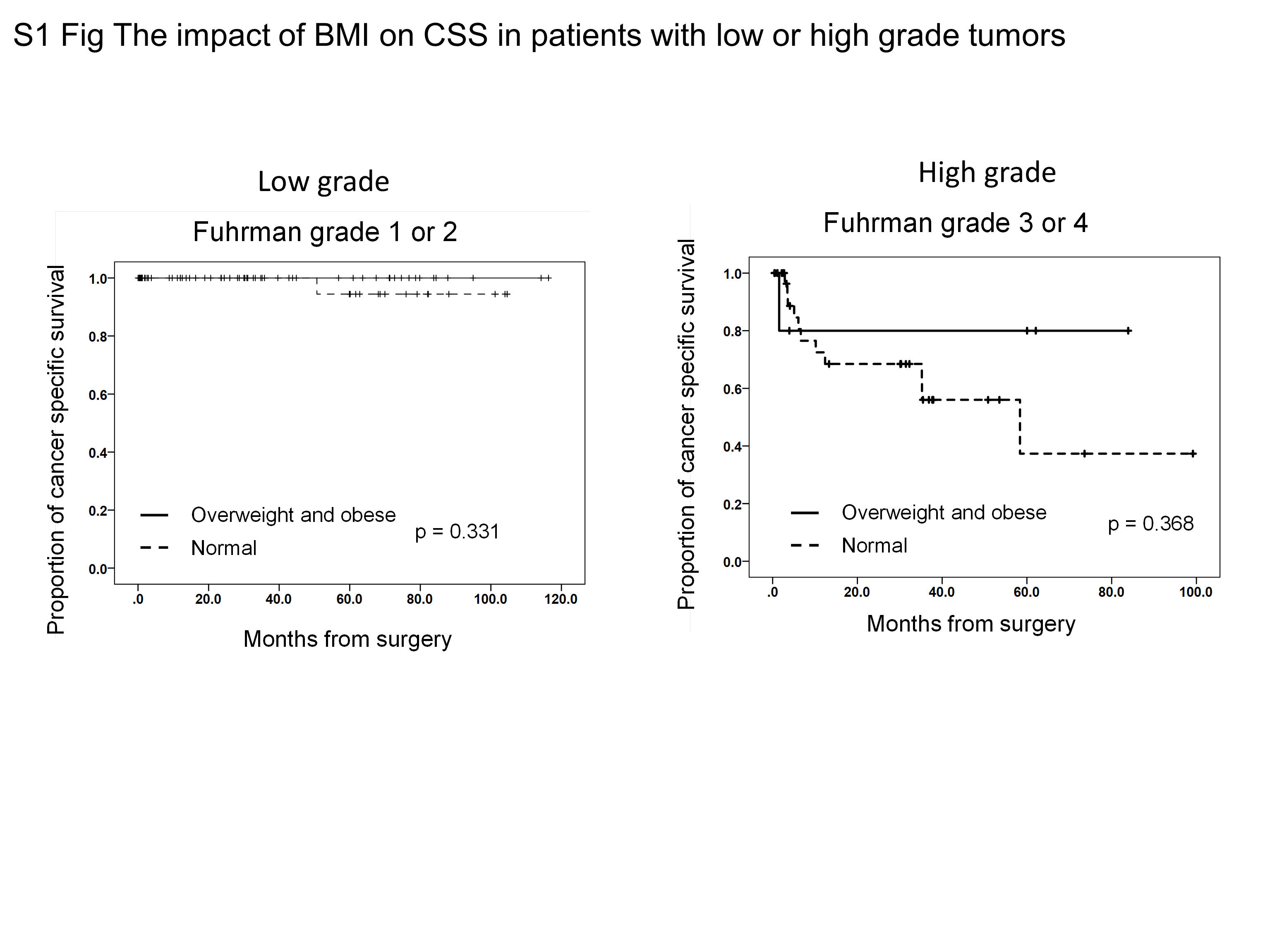

Supplement: S1 Fig — (TIF) [file pone.0171615.s001.TIF]

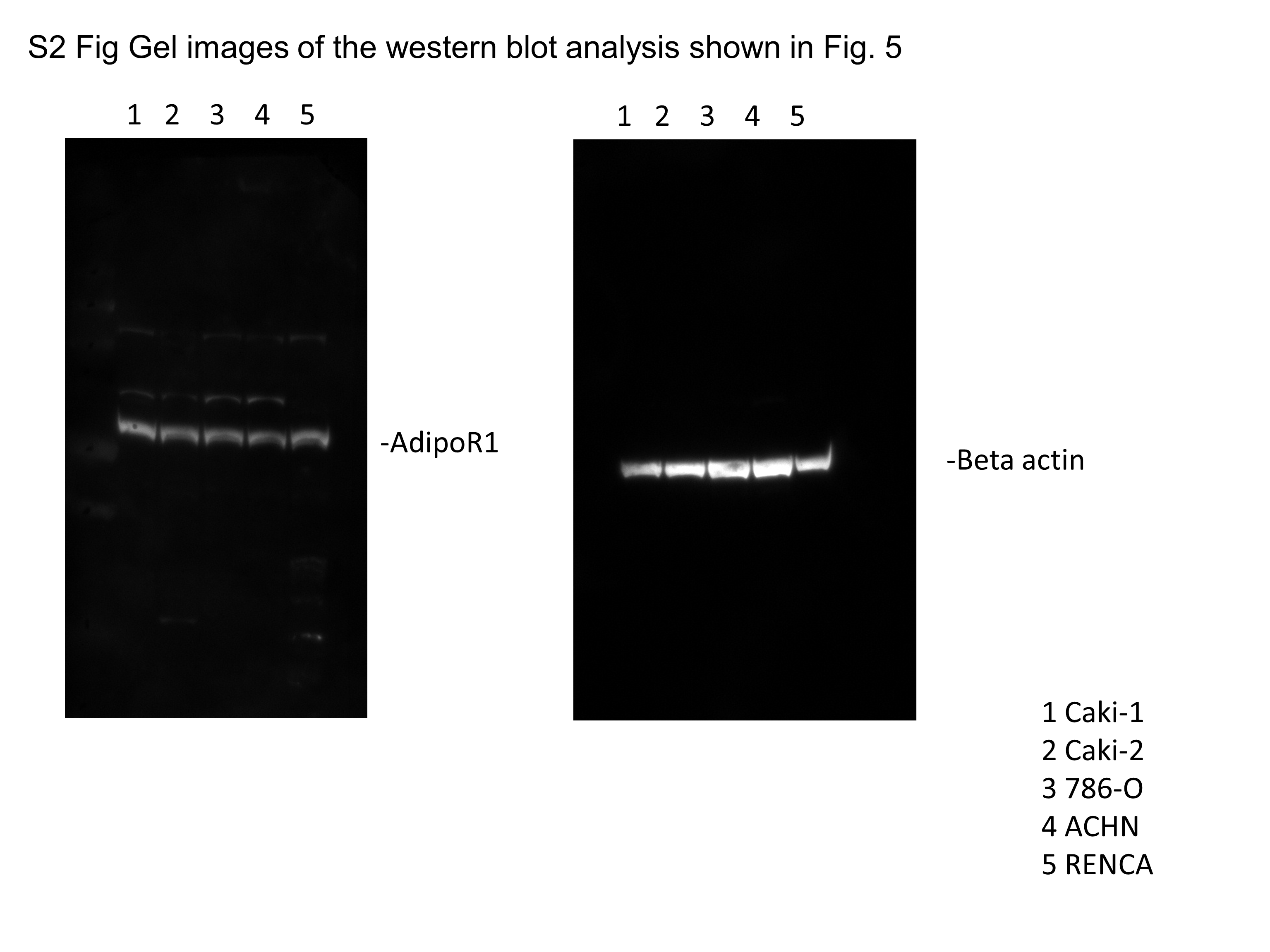

Supplement: S2 Fig — (TIF) [file pone.0171615.s002.TIF]

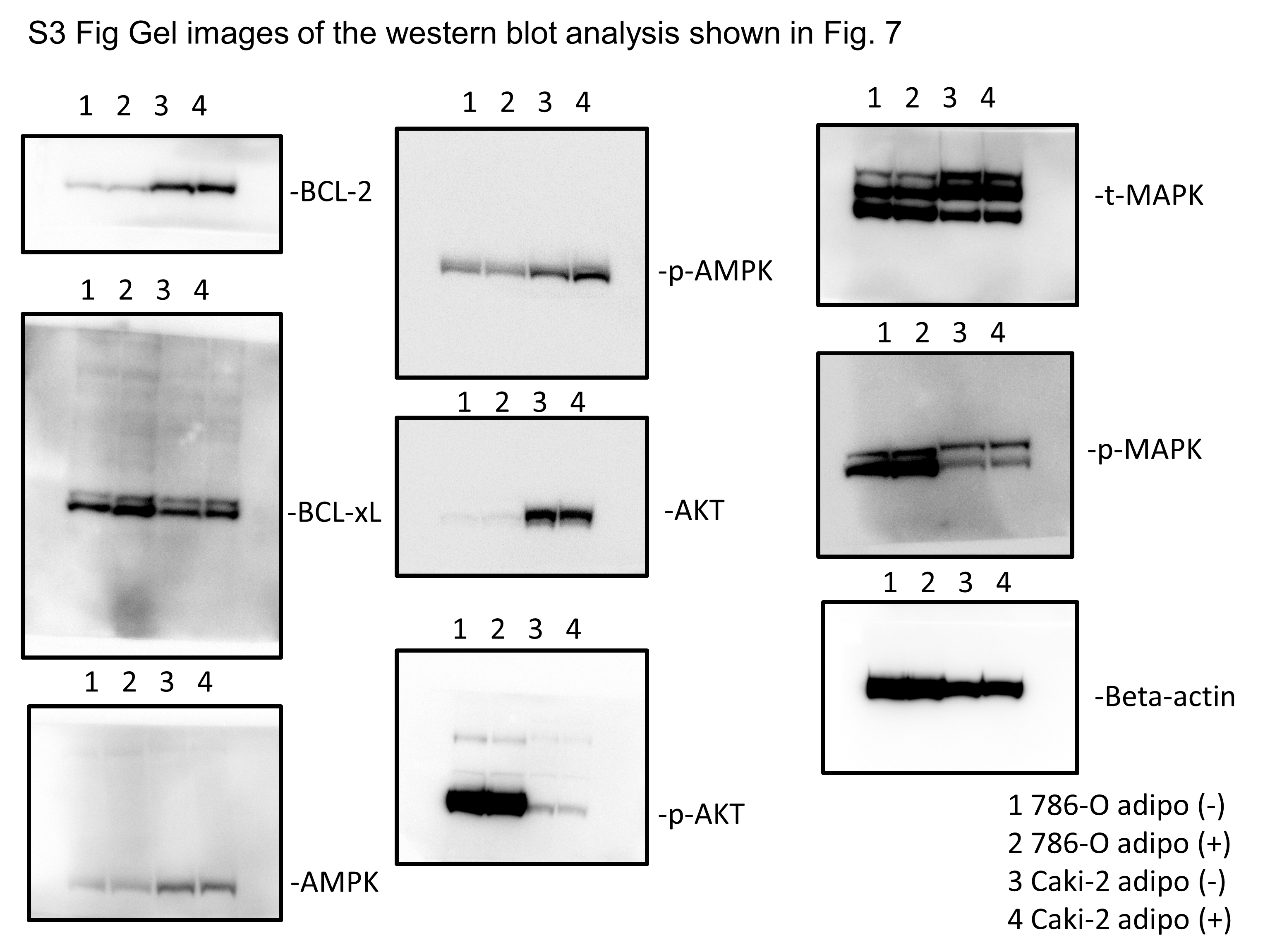

Supplement: S3 Fig — (TIF) [file pone.0171615.s003.TIF]
